# 

**Published:** 1908-11

**Authors:** 


					﻿Sixty-fourth Year.
Vol. LX1V. NOVEMBER, 1908.	No. 4
BUFFALO
MEDICAL JOURNAL
_______ XTW
AUSTIN FLINT	d.Hcfg. *
NOV. ‘*4. -79C8 EDITOR:
WILLIAM WARREN POTTER, M. D.
ASSISTANT EDITORS:
WILLIAM C. KRAUSS, M. D.	NELSON W. WILSON, M. D.
ASSOCIATE EDITORS:
JAMES WRIGHT PUTNAM, M. D. ERNEST WENDE, M. D.
JOHN PARMENTER, M. D.	JOHN A. MILLER, Ph. D.
MAUD J. FRYE, M. D.
				

